# The use of selected herbal preparations for the disinfection of Japanese quail hatching eggs

**DOI:** 10.1016/j.psj.2022.102066

**Published:** 2022-07-26

**Authors:** Karrar I.A. Al-Shammari, Justyna Batkowska, Magdalena Gryzińska, Łukasz Wlazło, Mateusz Ossowski, Bożena Nowakowicz-Dębek

**Affiliations:** ⁎ Department of Animal Production Techniques, Al-Musaib Technical College, Al-Furat Al-Awsat Technical University, Babylon, Iraq; † Institute of Biological Basis of Animal Production, University of Life Sciences in Lublin, 20-950, Lublin, Poland; ‡ Department of Animal Hygiene and Environment, University of Life Sciences in Lublin, 20-950, Lublin, Poland

**Keywords:** hatchability, ginger, garlic, oregano, cinnamon

## Abstract

The aim of study was to evaluate microbial and hatchability traits as well as chicks quality after hatching eggs disinfection with aqueous solutions of ginger (**GR**), garlic (**GC**), oregano (**O**), and cinnamon (**C**) extracts. The experiment was divided into 2 stages, at preliminary in vitro stage antimicrobial susceptibility of plant extracts (**PE**s) was tested against reference strains from the American Type Culture Collection. O and GC extracts had the best antimicrobial properties (*P* < 0.05). Then in in vivo stage 2,400 Japanese quails hatching eggs were divided into 6 groups, 400 eggs each. Eggs from first group were not disinfected (NC, negative control), eggs from second group were disinfected by formalin fumigation (C, positive control), in other groups 5% aqueous solutions of plant extracts of GR, GC, O, C were applied by spraying respectively. After standard incubation fertility, hatchability and periodical embryonic mortality were calculated as well as the body weight and livability of chickens during 14 d of rearing. Egg disinfection by aqueous solution of PEs led to maintain the hatchability, chick weight at hatch and post hatch body weight and early mortality of birds. Exclusion of any fungal isolates on eggshell surface was induced by GC followed by O and C groups (*P* < 0.05). In case of the bacteria colonies reduction only GR extract was effective. Chosen plant extracts may be treated as safe and alternative substances to traditional disinfectants of hatching eggs.

## INTRODUCTION

Egg is an ideal environment for the embryo development but also for the micro-organisms development. At the time of laying, the number of bacteria on the egg shell may range from 4.0 to 4.5 log CFU/eggshell (Reu et al., 2008). Eventually, harmful microorganisms penetrate the shell and infect the avian embryo, causing losses in hatchability, poor quality of chicks, and infection in growing birds. Copur et al. (2010) mentioned that the hatching eggs are infected by numerous infectious organisms before and after laying. Among them *E. coli, Proteus* spp., *Enterobacter* spp., *Pseudomonas* spp., *Klebsiella* spp., *Staphylococcus* spp., *Streptococcus* spp., *Clostridium* spp., *Bacillus cereus, Salmonella typhimurium, Micrococcus* spp., *Enterococcus* spp., molds and yeasts are the most common microorganisms that have been isolated from hatching eggs. Therefore, the shell cleanness and the effectiveness of its disinfection is critical to achieve a high level of hatchability, reducing the burden of pathogens and ensuring the production of high quality chicks (Reu et al., 2008).

The most practical way of hatching eggs disinfection is still fumigation with formaldehyde gas (CH_2_O), prior to egg incubation (Cadirci, 2009). However, the recent studies have shown that the use of formaldehyde gas in hatchery practice is dangerous for workers as irritant to the eyes and the nose, has a lingering noxious odor, carcinogenic effect like nasopharyngeal cancer and leukemia and venting of its vapors is difficult (Whistler and Sheldon, 1989; Debes and Basyony, 2011). Thus, alternative disinfectants are needed to replace formaldehyde by nonchemical substances as an effective, safe and nontoxic natural hatching egg sanitization. Alternative methods of disinfection application in hatchery include various disinfectants like a chemical substances and physical agents for example, Virkon (Gholami-Ahangaran et al., 2016), UV light (Al-Shammari et al., 2017), chlorine dioxide (Kim et al., 2016), ethanol (Nowaczewski et al., 2013), colloidal silver (Batkowska et al., 2017), H_2_O_2_ (Cox et al., 1999), ozone (Hrnčár et al., 2012; Wlazlo et al., 2020) or more natural substances like propolis (Batkowska et al., 2018a) or red grapefruit juice (Batkowska et al., 2018b). Among them are also plant extracts, to elimination microbial contamination and improve or keep at least the hatchability of chicks within normal limits (Samberg and Meroz, 1995).

It has been found that plant extracts (**PE**s) activity probably depends on pH, chemical composition, concentration or the specific bioactive compounds, also on population and types of affected pathogens. Christaki et al. (2012) stated that specific mode of action of essential oils belong to their components which include: monoterpenes, phenylpropene, and nonphenolic secondary metabolites which have a variable antimicrobial capacity and are important to eliminate any microbial contamination. Using plant extracts as disinfectants for hatching egg is due to their antimicrobial characteristics which belong to the diverse chemical bioactive compound mainly concentrated in essential oils of plants (Yildirim et al., 2003; Copur et al., 2010) .

The aim of study was to evaluate microbial and hatchability traits as well as chicks quality after hatching eggs disinfection with aqueous solutions of ginger (**GR**, *Zingiber officinale*), garlic (**GC**, *Allium sativum*), oregano (**O**, *Origanum vulgare*), and cinnamon (**C**, *Cinnamomum verum*) extracts.

## MATERIALS AND METHODS

The research was conducted with the approval of the Local Ethical Committee at the University of Life Sciences in Lublin (No. 16/2014). The commercial, chemically pure powdered plant extracts of ginger (GR, *Zingiber officinale*), garlic (GC, *Allium sativum*), oregano (O, *Origanum vulgare*), and cinnamon (C, *Cinnamomum verum*) were used. The content of chosen phytochemical in PEs were analyzed as it was described before (Al-Shammari et al., 2019).

The PEs were used as powdery extracts, which was dissolved in distilled water by volumetric flask to produce 5% concentration. The experiment was divided into 2 stages. At preliminary stage the antimicrobial susceptibility of PEs was tested. The in vitro antimicrobial activity of the prepared plant extracts was tested against reference strains from the American Type Culture Collection (ATCC): *Staphylococcus aureus* ATCC 25923, *Staphylococcus epidermidis* ATCC 12228, *Bacillus cereus* ATCC 10876, *Proteus mirabilis* ATCC 12453, *Escherichia coli* ATCC 25922, *Pseudomonas aeruginosa* ATCC 27853 and *Salmonella typhimurium* ATCC 13076. Fresh 24-h cultures on Tripticase Soy Agar (**TSA**) were used in this study.

Tests were performed using the microdilution method in Mueller-Hinton Broth broth (**MHB**) in 96-well microtiter plates. Minimum inhibitory concentration (**MIC**) and minimum bactericidal concentration (**MBC**) were evaluated. First, solutions of 200 mg/mL were prepared by dissolving individual plant extracts in dimethyl sulfoxide (DMSO). Then, a basic solution with a concentration of 40 mg/mL of the test compounds in MHB medium was prepared. Using the previously prepared solution, serial twofold dilutions were made in MHB obtaining extract concentrations ranging from 40 to 0.02 mg/mL.

Bacterial inoculum (with an optical density of 0.5 McFarland) was prepared in sterile saline from each strain. Then, 2 µL of bacterial suspensions were transferred to the wells each, obtaining a concentration of approximately 10^6^ CFU/mL. In addition, in the last 2 wells, a positive control (inoculated with bacterial suspension only) was performed to check the viability of the strain and a negative control (medium alone, without inoculum added) was performed to check the sterility of the medium.

The MIC value was determined by observing the lowest concentration at which no bacterial growth was observed in the well after incubation at 36 to 37°C for 24 h. Spectrophotometric method was used for this purpose by reading the absorbance at 570 nm using EL × 808 microplate reader (Bio-Tek Instruments, Inc.). For the determination of MBC, 5 µL of the contents of each well were transferred onto Mueller-Hinton Agar medium (**MHA**). The media were incubated at 36 to 37°C for 18 to 24 h. After incubation, MBC concentrations at which no bacterial growth occurred were determined. MBC/MIC ≤4 was taken as bactericidal effect and MBC/MIC >4 as bacteriostatic effect. The tests were performed in triplicate.

The main stage of experiment was performed in vivo. The materials consisted of 2,400 fresh hatching eggs (weight about 10.5 g) obtained from Japanese quailslayers at the age of 14 wk. The quails were housed in battery cages system with sex ratio (1♂:4♀) and fed with a breeder diet containing 2,900 kcal of metabolizable energy/kg and 20% of crude protein with free access to feed and water. Eggs were collected twice a day (in the morning and evening), taking into consideration that dirty eggs (fecal–contaminated eggshells) and eggs with visible cracks of shell were discarded as hatching eggs. After collection, eggs were stored for no longer than 3 d at 15 to 18°C and 75% relative humidity before the start of research.

Eggs were divided randomly into 6 groups before incubation, 400 eggs per group (4 replication subgroups in each). Eggs from first group were not disinfected (NC, negative control), eggs from second group were disinfected by fumigation with formaldehyde gas composed of 21 ml formalin (40%), 17 g KMnO_4_ and 21 mL of water (PC, positive control). For disinfection in groups third, fourth, fifth, and sixth, aqueous extracts of GR, GC, O, and C were used respectively (5% distilled water solution).

Prior to eggs placing in the incubator, eggs were numbered according to sanitization treatments and disinfected by each aqueous extract of PEs using hand sprayer and covering the whole egg surface with solutions. After spraying, the egg trays were kept outside the incubator for 15 min at room temperature (24°C) to dry after disinfection procedures. With respect to egg fumigation by formaldehyde gas, it was implemented inside special chamber.

Eggs were hatched artificially using a Jarson hatching apparatus (Jarson, Gostyń, Poland) under standard conditions of incubation:

- setting compartment–37.6 to 38.0°C temp. and 50 to 65% relative humidity.
- hatching compartment–37.0 to 37.5°C temp. and 75 to 80% relative humidity.

Eggs in setting compartment were turned automatically through 90° every 3 h (8 times a day). On 14th d of incubation the eggs were candled to determine the number of fertile and infertile eggs and dead embryos and moved from the setter to the hatching compartment. Fertility was calculated as the percentage of set eggs. Also, all eggs including fertile, infertile and unhatched eggs and hatched chicks were weighed and eggshell conductance constant (*K*) was determined as criterion of egg weight loss (moisture loss from egg) using the formula of Christensen et al. (2001). Also on 14th d the samples of eggs for microbial analyses were collected. The microbial evaluation was done according methods described previously (Batkowska et al., 2017, 2018a, b). Briefly, 10 eggs per each group were placed in sterile boxes contained 50 mL of phosphate-buffered saline (**PBS**) with 3 drops of TWEEN 80. Containers with eggs were left on the stirrer for 1 h. Samples were serially diluted in PBS and plated on sterile medium in order to obtain the total number of aerobic mesophilic bacteria, the total number of bacteria, coliform bacteria, hemolytic bacteria, *Salmonella* spp., *Staphylococcus* spp., yeast, and mold fungi. After incubation colonies were counted and presented as cfu/1 mL of liquid from the egg. To identify the bacterial colonies, a microscopic examination was performed as well as Gram's staining method and API biochemical tests (bioMérieux Polska). Molds were identified using special keys (Fassatiova, 1983; Watanabe, 2002).

After 17.5 d of incubation the number of hatched chicks, dead embryos, healthy and crippled chicks was recorded to determine the hatchability and mortality for both fertile and set eggs. Chicks were individually identified with numbered leg ring and raised (4 pens/group) according to their eggs disinfection treatments. They were reared under standard conditions of cage system for 14 d (Regulation of the Ministry of Agriculture and Rural Development dated on 28 June 2010, Poland), a balanced diet (CP 22.72, ME 2702.5) was provided *ad libitum*. The body weight (**BW**) and survivability of chicks were registered on 1st, 7th, and 14th d of their age.

The data were analyzed with the use of statistical package SPSS 24.0PL (IBM SPSS, 2016). The normality of data was verified using Kolmogorov-Smirnov test. The significance level was defined as 5% (*P ≤ 0.05*). The one-way ANOVA with Tukey's test was carried out. The mortality, hatchability and bacterial number of colony forming units were verified using nonparametrical χ^2^ test. All of the used tests were mentioned in tables.

## RESULTS

Table 1 presents the results of phytochemicals analysis in particular plant extracts. Cinnamon extract appeared to be the most rich in essential oils, whereas ginger extract contained the highest amount of flavonoids.

**Table 1:** Chemical composition of some phytochemicals in powdered plant extracts.

Phytochemicals	GR	GC	O	C
Essential oil (%)	0.10	0.45	0.30	1.25
Flavonoids (%)	0.0035	0.0023	0.0023	0.007
O-dihydroxyphenols (%)	0.214	0.071	0.705	0.278
Valerenic acid (%)	0.0047	0.0128	-	-
Glucosinolates (µmol/g)	0.031	-	0.004	-

C, cinnamon; GC, garlic; GR, ginger; O, oregano.

As indicated in the Table 2, the MIC values against Gram-positive bacteria for all tested extracts were 20 mg/mL, while showing bactericidal activity (MBC/MIC ≤4). The oregano and garlic extracts had the best antimicrobial properties. For oregano extract, the MBC/MIC ratio was 1 (MIC = 20 mg/mL, MBC = 20 mg/mL) for all Gram-positive bacteria. Only for garlic extract the MBC/MIC was >2 (MIC = 20 mg/ML, MBC ≥ 40 mg/mL) for *B. cereus* ATCC 10876. The highest bactericidal activity of all tested extracts was observed against *S. epidermidis* ATCC 12228 (MBC/MIC = 1). Gram-negative bacteria were more sensitive to the tested extracts compared to Gram-positive bacteria. MIC values ranged from 10 to 20 mg/mL. All extracts showed bactericidal activity (MBC/MIC ≤4), except cinnamon extract, which showed bacteriostatic activity (MBC/MIC > 4) against *S. typhimurium*. The most sensitive Gram-negative bacterium was found to be *P. aeruginosa* ATCC 27853 with MIC = 10 mg/mL for all extracts tested.

**Table 2:** MIC (mg/mL) and MBC (mg/mL) values of analysed plant extracts against reference strains.

Microorganisms	GR			GC			O			C		
	MIC	MBC	MBC/MIC	MIC	MBC	MBC/MIC	MIC	MBC	MBC/MIC	MIC	MBC	MBC/MIC
Gram-positive bacteria												
*Staphylococcus aureus*ATCC 25923	20	>40	>2	20	20	1	20	20	1	20	>40	>2
*Staphylococcus epidermidis*ATCC 12228	20	20	1	20	20	1	20	20	1	20	20	1
*Bacillus cereus*ATCC 10876	20	>40	>2	20	>40	>2	20	20	1	20	>40	>2
Gram-negative bacteria												
*Proteus mirabilis*ATCC 12453	20	40	2	20	40	2	20	40	2	20	40	2
*Escherichia coli*ATCC 25922	20	40	2	20	40	2	20	20	1	20	40	2
*Pseudomonas aeruginosa*ATCC 27853	10	20	2	10	20	2	10	20	2	10	20	2
*Salmonella typhimurium*ATCC 13076	20	40	2	20	>40	>2	20	40	2	10	>40	>4

C, cinnamon; GC, garlic; GR, ginger; O, oregano.

Depending on group, Table 3 shows that lack of differences was showed in fertility, hatchability, mortality (15–17.5 d), total mortality on fertile eggs and crippled chicks. The lowest mortality (0–14 d) of set eggs (*P = 0.001*) and fertile eggs (*P = 0.011*) was recorded by NC and low total mortality on set eggs (*P = 0.043*) was in PC. All disinfectant groups and NC did not significantly differ in eggshell conductance const. (*K*) value and healthy chick proportion in egg. In weight loss of fertile eggs GR, GC and O did not differ from NC and PC in this trait. However, disinfectant solution of C led to positive reduction of weight loss of fertile eggs (%).

**Table 3:** Hatchability traits of Japanese quails influenced by eggs disinfection with aqueous solutions of plant extracts.

Traits		Groups						
		NC	PC	GR	GC	O	C	*χ^2^ (p-value)*
Fertility		73.13	68.28	88.59	85.81	88.89	83.33	*0.566*
Hatchability	Set eggs	59.38	57.93	63.76	62.84	73.86	67.33	*0.796*
	Fertile eggs	81.20	84.85	71.97	73.23	83.09	80.80	*0.940*
Mortality0 – 14 days	Set eggs	2.50	5.52	14.77	14.19	9.15	6.00	*0.001*
	Fertile eggs	3.42	8.08	16.67	16.54	10.29	7.20	*0.011*
Mortality15 – 17.5 days	Set eggs	11.25	4.83	10.07	8.78	5.88	10.00	*0.369*
	Fertile eggs	15.38	7.07	11.36	10.24	6.62	12.00	*0.356*
Total mortality	Set eggs	13.75	10.34	24.83	22.97	15.03	16.00	*0.043*
	Fertile eggs	18.80	15.15	28.03	26.77	16.91	19.20	*0.242*
Crippled chicks (% of hatched chicks)		0.00	0.00	0.00	0.00	0.00	0.00	*-*
								SEM
Eggshell conductance constant – *K*	Infertile eggs	0.217	0.187	0.177	0.335	0.112	0.281	*0.024*
	Dead embryos	0.136	0.196	0.215	0.359	0.212	0.203	*0.024*
	Fertile eggs	0.151^ab^	0.208^a^	0.182^ab^	0.207^ab^	0.165^ab^	0.111^b^	*0.009*
	Unhatched eggs	0.196	0.237	0.215	0.254	0.214	0.216	*0.015*
Weight loss (%)	Infertile eggs	14.65	12.65	11.94	22.64	7.57	19.00	*1.606*
	Dead embryos	9.18	13.25	14.52	24.28	14.37	13.73	*1.608*
	Fertile eggs	10.01^a^	14.07^a^	12.29^a^	28.86^a^	11.16^a^	7.51^b^	*1.467*
	Unhatched eggs	16.54	20.04	18.14	21.47	18.10	18.23	*1.227*
Healthy chick (% of egg)		70.98	67.19	69.86	70.78	66.29	62.09	*0.910*

C, cinnamon; GC, garlic; GR, ginger; NC, negative control; O, oregano; PC, positive control.

a,b Means within rows (for groups) differ significantly at *P ≤ 0.05*

Table 4 revealed the microbial counts of egg shell under disinfectants effect. The O and C did not differ from NC in total number of fungi but decreased from PC whereas GR did not differ from PC. In GC, no fungal colonies were found. In total number of bacteria, GR did not differ from PC and NC but O, GC, and C had high counts. In terms of identified bacteria species, *E. coli* in GC, *Salmonella* spp. in (GC, O, C), *Staphylococcus aciuri* in (PC, GR, GC, O, C), *Staphylococcus epidermidis* in (PC, GR, O), *Staphylococcus* spp. and nonidentified bacteria in (GC and O) have not been detected.

**Table 4:** Microbial counts on egg shell of Japanese quails influenced by eggs disinfection with aqueous solutions of plant extracts.

Traits	Groups									
	NC	PC		GR		GC		O	C	SEM
Total number of fungi*	0.96^b^	1.36^a^		1.18^a^		0.00		1.00^b^	0.92^b^	*0.285*
Total number of bacteria*	1.67^c^	1.53^c^		1.70^c^		2.17^b^		2.55^a^	2.14^b^	*0.062*
Identified bacteria species⁎⁎										*χ^2^ (P-value)*
*E. coli*	9.80	14.6		10.6		0.00		0.80	8.10	*0.025*
*Salmonella* spp.	7.60	8.30		23.4		0.00		0.00	0.00	*0.000*
*Staphylococcus aciuri*	44.6	0.00		0.00		0.00		0.00	0.00	*0.000*
*Staphylococcus epidermidis*	13.0	0.00		0.00		40.0		0.00	5.40	*0.000*
*Staphylococcus* spp*.*	5.40	20.8		31.9		0.00		0.00	14.9	*0.000*
*Streptococcus* spp*.*	17.4	50.0		14.9		60.0		99.2	60.8	*0.000*
Non identified bacteria	2.20	6.30		19.1		0.00		0.00	10.8	*0.002*

C, cinnamon; GC, garlic; GR, ginger; NC, negative control; O, oregano; PC, positive control.

a,b Means within rows (for groups) differ significantly at *P ≤ 0.05*.

⁎ Log_10_ CFU/1ml of liquid from egg.

⁎⁎ % of total isolates.

No differences between GR, GC, O were stated as well as between NC and PC in body weight at hatch and at 14th d. Also, GR and GC did not differ from PC and NC with respect to BW at seventh day. However, C reduced BW at hatch and 14th d and O and C reduced BW at seventh day. Lack of differences in mortality and survivability in post hatch depending on group was recorded (Table 5).

**Table 5:** Body weight and mortality of Japanese quails influenced by eggs disinfection with aqueous solutions of plant extracts.

Traits		Groups						
		NC	PC	GR	GC	O	C	SEM
Body weight of birds at								
Hatch		6.93^a^	6.80^ab^	6.89^a^	6.66^ab^	6.78^ab^	6.28^b^	*0.061*
7th day		19.82^ab^	19.20^ab^	20.70^a^	18.31^b^	15.88^c^	14.53^c^	*0.290*
14th day		42.35^ab^	42.96^ab^	44.06^ab^	44.54^a^	39.92^b^	32.27^c^	*0.565*
Mortality (%)								*χ^2^ (P-value)*
1–7 d posthatch		3.16	2.38	1.05	0.00	0.00	3.16	*0.190*
8–14 d posthatch		0.00	0.00	0.00	0.00	0.00	0.00	*-*
Survivability (%)1–14 d posthatch		96.84	97.62	98.95	100.00	100.00	96.84	*0.190*

C, cinnamon; GC, garlic; GR, ginger; NC, negative control; O, oregano; PC, positive control.

a,b Means within rows (for groups) differ significantly at *P ≤ 0.05*

## DISCUSSION

All disinfectant solutions of PEs maintained stably the hatchability, total mortality on fertile egg, weight loss (%) of eggs, and embryos and healthy chick (%) as well as survivability of birds in post hatch. The C solution disinfectant exerted a potential effect to reduced water loss and *K* values of fertile eggs compared to NC and PC. It can be reasoned that C increased the amount of metabolic water in embryonic tissues through oxidizing energy-containing substances found in C and also for its antioxidative properties to protect the growing embryo tissues (Gul and Safdar, 2009). Egg weight loss is an important proxy for incubation and hatching success. Too fast moisture loss is unsuitable for normal embryonic development and metabolic status (Yildirim et al., 2003). The variation in the ability of embryos to adjust their water contents and eggshell conductance is essential to estimate the link between egg weight loss and embryonic survival (Shahein and Sedeek, 2014). The high *K* value due to egg treatment with CH_2_O vapor is reasonable since this disinfectant might affect the cuticle layer and shell porosity. The statistical stability of healthy chick ratio and BW at hatch for PC and NC compared to solution disinfectants of PEs is probably indicated to increase yolk sac weight in relation to embryonic mass and malabsorption of yolk materials and fatty acids contents by embryo (Şahan et al., 2014) whereas the PEs groups efficiently utilized yolk contents and decrease it in relation to embryonic tissues. Although this stability of BW was continuous until 14 d and also without influence on changing mortality. Copur et al. (2010) declared that hatchability of fertile eggs, discarded chicks rate, BW at hatch, BW gain, carcass quality, and feed intake (**FI**) up to 6 wk of age were not influenced by spraying hen eggs with 0.55 or 0.75 μL of O/cm^3^ for 2 exposure times, 3 and 6 h compared to formalin and NC.

The GC contributed to removing the fungi, due to sulfur containing compounds in GC, which showed antimicrobial activity against several molds and fungi. These natural compounds are active against *Candida albicans, Aspergillus flavus, Aspergillus niger, Curvularia lunata, Cryptococcus neoformans*, Trichophyton, Epidermophyton, Microsporum, and other fungi (Suleiman and Abdallah, 2014). Tsao and Yin (2001) proved that diallyl sulfide (**DAS**), diallyl disulfide (**DADS**), diallyltrisulfide (**DAT**), and diallyltetrasulfide (**DATS**) in GC oil had a powerful antifungal activity in vitro against 3 *Candida spp*. (*C.albicans, C. krusei*, and *C. glabrata*) and 3 *Aspergillus spp.* (*A. niger, A. flavus*, and *A. fumigarus*) and antimicrobial capability of these sulphides were determined by disulfide bonds. Allicin in GC was found to antimicrobial agent against wide range of strains of *E. coli, C. albicans* fungus, and intestinal protozoan parasites, such as *Entamoeba histolytica* and *Giardia lamblia* parasites (Ankri and Mirelman, 1999). In addition, antifungal activity of crushed GC also depends on various biologically active compounds during degradation, such as polysulfanes and vinyldithiins (Borlinghaus et al., 2014). Differently to our result was recorded by Copur et al. (2011), who stated that immersed eggs by an aqueous extract of allicin (major compound in GC) did not change the total bacteria, yeast, and mold on egg surface compared to PC.

Interestingly, in current data, O and C groups performed a better antifungal activity than PC and lack of most dangerous bacteria (*E. coli, Salmonella* spp., *Staphylococcus*, and nonidentified species) in eggshell as % of total isolates in most of PEs disinfectant solutions. It was also thought that the chemical structure, such as the existence of the functional hydroxyl (–OH) group attached to a phenyl ring in phenolic compounds of secondary metabolites in essential oils (**EO**s) of PEs have the greatest antimicrobial activity. Probably, its aromatic specificity is also responsible for the disruption of cell homeostasis, leading to growth suppression and cell death (Christaki et al., 2012). The superiority of GC, O, and C disinfectants in the total number of bacteria on shell eggs might arise from the natural variability of the composition in secondary metabolites of plant extracts.

Data contradictory to ours was reported by Yildirim et al. (2003), who proposed a decrease in the total bacterial population and without effect on fungi and coliforms on eggshell of quail disinfected by alcoholic extract of EOs extracted from O in comparison to PC. The same was reported by Copur et al. (2010), who stated that disinfection of fertile eggs by EOs of O decreased bacteria, yeast and mold populations on eggshell but the exposure times (3 and 6 h) in a fumigation cabinet at 24°C or the used doses of this extract did not affect microbial activity, compared to NC and PC. Also, Ulucay and Yildirim (2010) found that the application of major bioactive compounds in EOs present in O (carvacrol or thymol) and in C (cinnamaldehyde) as alcoholic disinfectants to quail eggshell has decreased fungi, coliforms and total bacteria counts compared to NC. Similarly to the current data, it was found that egg disinfected by EOs alcoholic extract of GR had no effect on fungi, coliforms and total bacteria, however, the same result was obtained by using EOs alcoholic extract of O or mixture (GR + O) compared to PC (Debes and Basyony, 2011).

## CONCLUSIONS

The plant extracts demonstrated various antimicrobial properties, however, the eggs disinfection by aqueous solution of plant extracts (ginger, garlic, oregano, cinnamon) led to maintain the hatchability, chick weight at hatch and post hatch body weight and early mortality of birds. Exclusion of any fungal isolates on eggshell surface was induced by garlic followed by oregano and cinnamon groups. In case of the bacteria colonies reduction only ginger extract was effective. Chosen plant extracts may be treated as safe and alternative substances to traditional disinfectants of hatching eggs.

## DISCLOSURES

All authors declare that they have no conflicts of interest.
